# Effect of Moderate Aerobic Exercise on Body Composition, Biochemical Parameters and Oxidative Damage in Older Women Without and With Metabolic Syndrome

**DOI:** 10.3390/jfmk11020169

**Published:** 2026-04-23

**Authors:** Liliana Gutiérrez-Lopéz, Ivonne María Olivares-Corichi, José Rubén García-Sánchez

**Affiliations:** 1 Laboratorio de Investigación en Ciencias Aplicadas al Deporte, Especialidad de Medicina de la Actividad Física y el Deporte, Escuela Superior de Medicina, Instituto Politécnico Nacional, Ciudad de México 11340, Mexico; lgutierrezl@ipn.mx (L.G.-L.); iolivares@ipn.mx (I.M.O.-C.); 2 Sección de Estudios de Posgrado e Investigación, Escuela Superior de Medicina, Instituto Politécnico Nacional, Ciudad de México 11340, Mexico

**Keywords:** aging, oxidative damage, aerobic exercise, metabolic syndrome

## Abstract

**Background:** Metabolic syndrome (MetS) is a cluster of pathologies (obesity, dyslipidemia, insulin resistance, hypertension) that affects over one quarter of old adults. MetS is a condition that markedly increases the susceptibility of various organs to dysfunctionality and is associated with the development of oxidative stress. The existing guidelines point out that exercise is highly advantageous for patients with MetS. However, there is a need for specific guidance and clinical evidence. **Objective:** This study aimed to investigate the effects of a moderate aerobic exercise program on older women without and with MetS. **Methods:** A total of 120 women aged 60–70 years old were recruited and divided into two groups: healthy old women (HOW, N = 60) and old women with MetS (OW-MetS, N = 60). Anthropometric values, biochemical parameters and markers of oxidative damage were evaluated before and after moderate aerobic exercise. Exercise was performed five days per week for three months (64 sessions). Each exercise session consisted of 40 min and included the following: (a) five minutes of warm-up exercise; (b) ten minutes of flexibility exercise with resistance using own weight and coordination; (c) twenty minutes of moderate-intensity aerobic exercise (heart rate max between 60% and 70%); and (d) five minutes to cool down/stretching with respiratory techniques. **Results:** A significant decrease in anthropometric variables was generated by the exercise program [waist circumference 4.35 cm (*p* < 0.05) in OW-MetS, body fat −1.55, −1.39% (*p* < 0.05) and muscle mass 0.8, 1.1% (*p* < 0.05) in HOW and OW-MetS, respectively]. The exercise program resulted in beneficial changes in all biochemical parameters in both groups. Importantly, HOMA values showed a significant decline of −0.85 and −6.17 in HOW and OW-MetS, respectively. Furthermore, oxidative stress was present in the OW-MetS group, which was reduced by the exercise program, resulting in a decrease in protein damage [formazan 45% and 42% in HOW and OW-MetS respectively] and an increase in antioxidant defenses (thiol groups 36%, 99% and GPx 55%, 20% in HOW and OW-MetS, respectively). **Conclusions:** The data of this study show that moderate aerobic exercise may be potentially useful in treating and preventing MetS in older patients.

## 1. Introduction

Metabolic syndrome (MetS) is a cluster of metabolic abnormalities that includes hypertension, abdominal obesity, insulin resistance and dyslipidemia [1]. However, this definition does not show the importance of each of the abnormalities in MetS, and different organizations have distinctly emphasized each of these abnormalities [2].

Because the prevalence of MetS increases with the advancing of age, as well as with sedentary behavior (insufficient amounts of weekly minutes (<150 min) of moderate-to-vigorous physical activity) [3], the risk of type 2 diabetes and the development of cardiovascular diseases are exacerbated in this population [4]. For this reason, the search for interventions that mitigate the progression of these metabolic disorders is highly relevant. In this context, exercise is an option that impacts cardiometabolic health [5]. Indeed, recent reports show that exercise has benefits for cognition, cardiometabolic health, and skeletal muscle quality and that it is an effective therapeutic strategy for patients diagnosed with MetS [5,6,7,8]. Trials using moderate-intensity and regular physical exercise demonstrated that these exercises confer many physiological health benefits [9,10,11]. However, exercise is a double-edged sword where muscle tissue consumes high amounts of oxygen and consequently generates reactive oxygen species (ROS), whose removal is necessary for normal cellular function. A high concentration of ROS causes oxidative damage to lipids, proteins, and DNA, which is related to cellular dysfunction, aging and a decrease in lifespan [12]. An imbalance between ROS and antioxidant defense creates a physiological state of oxidative stress (OS), which is related to the development of different pathologies [13]. Interestingly, the pathologies included in MetS induce OS, generating morphological alterations in the mitochondria, as well as a dysfunction in oxidative phosphorylation that induces mitophagy and apoptosis [14]. Because exercise has diverse effects on diseases, including an increase in serum antioxidant capacity [15,16], exercise is considered one of the non-pharmacological interventions for treating patients with MetS [17,18]. Although several guidelines indicate exercise for treating patients with MetS, there is a lack of specific guidance (frequency, timing and type of exercise) that shows clinical evidence of improvements in MetS. Despite the benefits of exercise, the design of programs and its effects on MetS in older adults remain unclear. Several studies have examined the effect of exercise on strength [19], frailty [20], bone density [21], mass and functional improvements in old adults [22]; this study shows the improvement in body composition, oxidative damage, lipid and metabolic profile. This study shows the effectiveness of the exercise prescription targeting metabolic health in old adults.

## 2. Materials and Methods

### 2.1. Participants

This study included participants from Integral Development of the Family (DIF, based on the Spanish acronym) at the “Frida Kahlo” rest house for women, located in the state of Mexico. Sixty healthy old women (HOW) and sixty old women with MetS (OW-MetS) were included in this study (60–70 years old). MetS was diagnosed based on the minimum criteria established by the World Health Organization (OMS) [23], International Diabetes Federation (IDF) [24], and Third Report of the National Cholesterol Education Program—Adult Treatment Panel III (NCEP–ATP III) [25]. This study was approved by the ethics and research committee of the School of Medicine at the National Polytechnique Institute (CI-01/19-09-2018). A written informed consent form was signed by all participants, and this study was conducted in accordance with ethical principles originating in the Declaration of Helsinki of 1975 as revised in 2013 and the Good Clinical Practice Guidelines. The exclusion criteria applied to this study were women who performed regular physical activity, smoked, and/or were diagnosed with any respiratory or cardiovascular diseases.

### 2.2. Blood Samples

Five milliliters of blood were obtained from the forearm (24 h before and 24 h after the intervention). Due to exercise causing a transient increase in biochemical parameters and oxidative stress, we decided not to take blood samples immediately after finishing the exercise program [26].

Blood was drawn into a test tube containing 86 USP of sodium heparin and was centrifuged at 3500 rpm for 15 min. Plasma was stored at −80 °C until needed. Plasma was used in the assessment of biochemical parameters, biomarkers of oxidative damage, and quantification of antioxidant defense.

### 2.3. Moderate Aerobic Exercise Program

The exercise program used in this study was designed to meet the adequate exercise and physical activity guidelines for older adults established by The American College of Sports Medicine (American College of Sports Medicine, 2017) [27]. The moderate aerobic exercise program was prescribed to the HOW and OW-MetS groups and was supervised personally by sports medicine physicians. The moderate aerobic exercise program was performed five days per week for three months (64 sessions). Each exercise session lasted 40 min and included the following: (a) five minutes of warm-up exercise (low-intensity and flexibility exercise); (b) ten minutes of flexibility exercise with resistance using own weight and coordination; (c) twenty minutes of moderate-intensity aerobic exercise (heart rate max between 60% and 70%; maximum heart rate was estimated using the formula (HR_max_ = 208 − 0.7 × age) [28], and it was monitored using an oximeter and a Polar thoracic heart rate band); (d) five minutes to cool down/stretching with respiratory techniques at the end of each section.

### 2.4. Exercise Adherence

Exercise adherence is defined as participation, duration, intensity, and completion of the exercise program [29]. In this study, all participants completed the exercise program and measurements (adherence was 100%). This was possible due to the exercise program being part of their physical activity during their stay in the rest house, and they attended daily.

### 2.5. Anthropometric Measurements

The following anthropometric parameters were determined before and after performing the moderate aerobic exercise program: age, size, weight, waist circumference, body mass index (BMI), percentage of lipid and muscle, and systolic and diastolic blood pressure. The body composition was determined with densitometric analysis using Brozek’s method [30].

### 2.6. Analysis of Biochemical Parameters

Biochemical parameters such as glucose, total cholesterol, and triglycerides were evaluated before and after the intervention involving the moderate aerobic exercise program. The triglyceride/high-density lipoprotein cholesterol (TG/HDL-C) ratio and the triglyceride–glucose (TyG) index were proposed as biomarkers for the identification of insulin resistance (IR) due to their correlation with the data of euglycemic–hyperinsulinemic clamps [31,32]. The TyG index was calculated with the following formula: [triglycerides (mg/dL) × glucose (mg/dL)]/2. Meanwhile, the formula for the homeostasis model assessment of IR (HOMA-IR) is as follows: fasting glucose (mg/dL)/fasting insulin (IU/mL) [33].

### 2.7. Plasma Biomarkers of Oxidative Damage

The status of oxidative damage in the study groups was determined in plasma. The lipoperoxidation products (lipid damage) assessed were malondialdehyde (MDA) [34], 4-hydroxynonenal (4-HNE) and thiobarbituric acid-reacting products (TBARSs) [35]. Briefly, MDA was determined using plasma (200 µL) diluted in H_2_O (1:1) mixed with a solution of 1-methyl-2-phenylindole (650 µL) in a mixture of acetonitrile/methanol (3:1). The reaction was then started by adding 150 µL of 37% hydrochloric acid. The absorbance at 586 nm was measured upon the incubation of the reaction mixture at 45 °C for 40 min. A blank sample in which the plasma was replaced with water was included.

4-HNE was determined following the procedure of MDA replacing hydrochloric acid by methanesulfonic acid containing 34 µM Fe^+3^ and measured after the incubation of the reaction mixture at 45 °C for 30 min. The final absorbance at 586 nm was a linear function of the concentrations of both MDA and 4-HNE. Therefore, the 4-HNE concentration was assessed after subtracting the MDA concentration.

TBARSs were determined by mixing 100 µL of plasma with 400 µL of buffer (Tris-Base 7.2 mM, pH = 8) and 1 mL of 0.375% thiobarbituric acid (TBA) in 0.2 N HCl. The mixture was incubated at 90 °C for 15 min and at room temperature for 5 min. Then, 500 µL of 0.2 N HCl was added, and the absorbance was measured at 529 nm. 1,1,3,3-Tetramethoxypropane (Sigma-Aldrich, St. Louis, MO, USA) was used as a standard. The data for MDA, 4-HNE, and TBARSs are expressed in micromoles (μM).

Carbonyl group quantification was utilized for assessing oxidative damage to proteins. The presence of carbonyl groups produced by the oxidation of phenylalanine and tyrosine amino acids (initial damage) was determined by mixing 200 µL of plasma and 2,4-dinitrophenylhydrazine (DNPH), which generated stable dinitrophenylhydrazone products detectable at 370 nm [36]. The molar extinction coefficient for DNPH (22,000 M^−1^ cm^−1^) was used to calculate the nmol of osazone. The data obtained were normalized by milligrams of protein and are expressed as osazone/mg protein.

The capacity of oxide proteins to react with nitroblue tetrazolium (NBT) to produce formazan was used to establish severe damage in proteins [37]. The molar extinction coefficient for formazan (E = 15 mM^−1^ cm^−1^) was used to calculate its concentration, which is expressed in nanomole (nmol) of formazan/mg protein. Lowry’s method was used to determine total proteins, which was used as a normalization parameter [38].

### 2.8. Quantification of Antioxidant Defenses

Thiol group concentration and the activity of glutathione peroxidase (GPx) are markers of antioxidant defenses. Total thiol groups were assessed in 100 µL of plasma mixed with 500 µL of Ellman’s reagent [39]. Then, the absorbance was determined at 412 nm, and the concentration of the thiol groups was calculated from the standard curve that was constructed with cysteine. The activity of GPx was evaluated by Lawrence and Burk’s method [40]. Briefly, plasma (100 µL) was mixed with 800 µL of redox solution, containing (mM) 50 KHPO_4_, 1 EDTA, 1 sodium azide, 0.2 NADPH, reductase glutathione 1 U/mL and reduced glutathione 1 mM, and incubated for 5 min at 37 °C. Finally, 0.25 mM H_2_O_2_ (100 µL) was added and mixed, and the absorbance was determined at 340 nm each minute for 3 min. Oxidized NADPH was calculated by multiplying the delta absorbance by the molar extinction coefficient for NADPH (E = 6.22 × 10^6^ M^−1^ cm^2^). The activity of GPx is expressed in µU/mg of proteins, where U denotes the µmol of oxidized NADPH per minute.

### 2.9. Statistical Analysis

Data is expressed as the mean ± SD. Student’s *t*-test was employed to analyze inter-group differences. Inter-group heterogeneity of the variables was tested using Levene’s F-test. The inter-group and inter-time interaction test was performed using two-way repeated measures analysis of variance. A probability value of ≤0.05 was statistically significant. Intra-group comparisons were performed using paired *t*-tests. All tests were conducted using the software Prism 8 (GraphPad, San Diego, CA, USA).

## 3. Results

### 3.1. Participants

A total of 120 older women (60 HOW and 60 OW-MetS) were included in this study. The participant characteristics are shown in Table 1. Both groups showed similar age and size; however, the OW-MetS group were overweight with high waist circumference, high body fat, low muscle mass, and hypertension (Table 1). In addition, the biochemical parameters of the OW-MetS group indicated the presence of hyperglycemia, triglyceridemia and insulin resistance (IR) according to HOMA (Table 1). All these parameters confirmed the presence of MetS according to the criteria established by the OMS, IDF and NCEP–ATP III.

In a pharmacological context, the participants were interviewed on medications used; only three participants were using acetaminophen in the HOW group (Table 2). As expected, medication for glycemic and hypertension control was used in the OW-MetS group. It is important to note that this medication did not interfere with the exercise interventions, and it remained unchanged throughout the intervention.

### 3.2. Biomarkers of Oxidative Damage

To evaluate oxidative damage in the study groups prior to the intervention, the biomarkers of oxidative damage in the plasma were assessed. Initially, all the biomarkers of the OW-MetS group were higher than those of the HOW group (Table 3). These data indicated the presence of oxidative damage in lipids and proteins. In addition, the antioxidant defense values (thiol groups and GPx activity) of the OW-MetS group were lower than those of the HOW group (Table 3). These data indicated the presence of oxidative stress in the OW-MetS group.

### 3.3. Effects of Moderate Aerobic Exercise Programs on Anthropometric Variables and Biochemical Parameters

A moderate aerobic exercise program was prescribed to the study population. The intervention and measurements were completed by all participants, and no one dropped out of the study. This was possible due to the participants being members of the rest house for women; they come every day and spend all day. This allowed personalized monitoring and consequently better control of the participants. In this context, the program generated a reduction in the waist circumference of the OW-MetS group; furthermore, this program showed positive effects on the percentage of fat and muscle in both groups (Table 4). Regarding biochemical parameters, the exercise program produced beneficial changes in all participants in both groups (Table 4).

### 3.4. Effects of Moderate Aerobic Exercise Programs on Oxidative Stress

**Figure 1:**
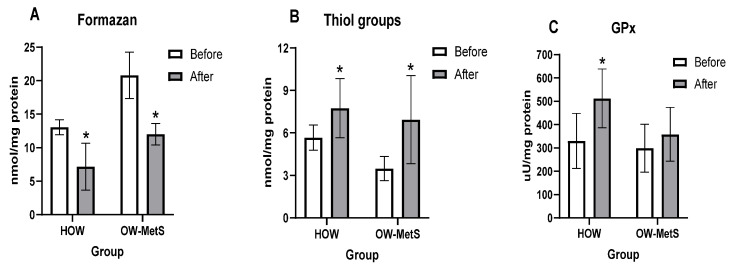
Effects of exercise program on oxidative damage and antioxidant defense. (**A**) A decrease in oxidative damage in proteins (formazan) and (**B**,**C**) increases in antioxidant defense (thiol groups) and GPx were generated after the exercise program in HOW and OW-MetS groups. Data are expressed as mean ± SD and analyzed by paired *t*-test * *p* < 0.05.

The effect of the exercise program on oxidative stress was analyzed after the intervention. Table 5 shows a decrease in oxidative damage in both groups, and all the changes in the oxidative damage markers, except for 4-HNE, in the OW-MetS group were statistically significant. Oxidative damage to proteins (based on the formazan biomarker) was detected in both groups, and it decreased with the exercise program; furthermore, an increase in antioxidant defenses (thiol groups and GPx) was detected in both groups (Figure 1). These data suggest that the exercise program generated redox homeostasis in the participants.

## 4. Discussion

This study reported the beneficial effects of a moderate aerobic exercise program on anthropometric measures, biochemical parameters, oxidative damage, and antioxidant defenses in old women without or with MetS.

Evidence shows that exercise is part of a treatment strategy for MetS. However, few studies provide specific guidance (pertaining to frequency, timing, and type of exercise) to impact the components of MetS [41,42,43]. In this study, a moderate aerobic exercise program was designed and prescribed as a strategy to treat MetS. In this sense, the exercise program improved waist circumference and generated a positive impact on the percentage of fat and muscles (Table 4). Furthermore, this intervention generated important changes in biochemical parameters: (1) it improved the lipidemic profile by raising HDL-c levels and lowering triglycerides, and (2) it increased insulin sensitivity (by decreasing glucose, insulin levels and HOMA-IR). Different biological pathways may explain these benefits; for example, it is reported that exercise increases HDL-C and in consequence cholesterol efflux through reverse cholesterol transport (RCT) [44]. In this context, plasma HDL-C formation is associated with increased expression of ATP-binding cassette transporter A-1 (ABCA1) in macrophages; that is, gene expression is modified after exercise [45]. On the other hand, liver X receptor (LXR) is a transcription factor involved in regulation of ABCA1 expression. Interestingly, LXR expression also is induced by the exercise; indeed, LXR improves the RCT process due to increased plasma HDL-C levels [46].

With reference to triglycerides, we found a favorable reduction that can be explained by the increased energy metabolism generated by higher muscular activity. In this case, the muscles have a function that leads to metabolic challenges; therefore, triglycerides are used as the primary source of energy in the muscle and are favored by the upregulation of lipoprotein lipase (LPL) activity [47]. In addition, when we consider that exercise increases the activity of hormone-sensitive lipases (HSLs) in adipose tissue and muscle, triglycerides are converted efficiently into free fatty acids and removed. In addition, it is important to mention that regular activity has three important effects that contribute to the metabolism of triglycerides: (a) it increases the activity of hormone-sensitive lipases (HSLs) in adipose tissue and muscle, allowing triglycerides to be more efficiently converted to free fatty acids; (b) it increases the expression of plasma membrane fatty acid-binding proteins, such as FABPPM, which allows that the fatty acids enter to muscle efficiently; and (c) it increases the intramuscular capacity to bind fatty acids in the cytosol and transport them to the mitochondria for β-oxidation [48].

The OW-MetS group showed elevations in glucose, insulin, and HOMA at baseline; however, the exercise program generated important benefits (Table 4). This may be attributed to an increase in muscle metabolic capacity, in which exercise induced muscle glucose uptake via GLUT-4 translocation and improved the insulin signal pathway [49]. This evidence highlights the benefits of a structured and supervised moderate aerobic exercise program for treating MetS. Although an exercise program is viewed as a non-pharmacological method of treating MetS, its effects observed in the HOW group suggest that it can be a lifestyle recommendation for preventing MetS (Table 4).

Noteworthily, accumulating evidence shows that exercise is key for modifying the clinical components of MetS; however, studies that show its impact on oxidative damage are limited [50,51,52]. In this regard, our study shows that the exercise program reduced oxidative damage in the HOW and OW-MetS groups, which could be explained by the increase in antioxidant defenses generated by the exercise program (Figure 1). Indeed, the evidence points to exercise as an activator of the erythroid-related nuclear factor 2 (NRF2) pathway (transcription factor responsible for the antioxidant response [53]); it is proposed that the exercise program could be related to NRF2 activation and the generation of a homeostasis redox, which explains the diminishing of oxidative damage (Table 5). This possibility is supported by the increase in GPX activity (Figure 1) and its regulation by NRF2 [54].

These data demonstrate that the imbalance between free radicals and antioxidants can be rectified by moderate aerobic exercise. Indeed, when we consider oxidative stress to be associated with inflammation, risk of cardiovascular diseases, and insulin resistance [16,55,56,57], a systemic redox balance could be responsible for the benefits generated. In this sense, the evidence shows that insulin is modified by reactive oxygen species, generating insulin that is not functional and related to insulin resistance [58]. Interestingly, insulin modification is prevented by a redox balance [59]; this balance generated by the exercise program interferes with insulin modification, which in turn favors the changes observed in glucose parameters (Table 4).

Noteworthily, evidence indicates that exercise and aging increase reactive oxygen species (ROS) production [60,61,62]; however, we propose that a structured exercise intervention (specific guidance, monitoring, and a better control of the participants) generates muscular adaptation to exercise and consequently significant health benefits. In this context, we detected that the HOW and OW-MetS groups showed severe oxidative damage to lipids and proteins (Table 5); nevertheless, the exercise program decreased this damage in both groups, which was evident from the increase in antioxidant defenses (thiol groups and GPx activity) (Figure 1). On this point, it is important to mention that the exercise program did not change the 4-HNE values in the OW-MetS group, which could be explained by the metabolic disorder present in this group. We propose a scenario in which impaired metabolic pathways of 4-HNE could be generating an increase in its levels in plasma [63,64,65,66,67]. Furthermore, when we consider that the OW-MetS group showed changes in insulin resistance but did not reach normal values (HOMA, Table 4), the existence of a strong inflammatory response is favored, in which interleukin (IL)-6 is the major inflammatory mediator. In this scenario, the inflammatory process contributes to increasing 4-HNE via the action of cyclooxygenase [66]. In this context and considering the changes generated in the other biomarkers of oxidative damage and antioxidant defense in the OW-MetS group, we postulate that a higher intervention time in this group could decrease insulin resistance, inflammation, and, in consequence, 4-HNE values. This is in agreement with reports by other authors on the impact of exercise on oxidative stress [68,69]. Moreover, as MetS is an inflammatory state that induces ROS production [70], in this study, the protection provided by the exercise program against oxidative damage confirms that it can be used in treating or preventing MetS.

This study demonstrates that moderate aerobic exercise modifies the MetS parameters. Furthermore, the exercise program reduced oxidative damage in the study groups, generating redox homeostasis. The study’s findings emphasize the therapeutic potential of exercise to mitigate or prevent MetS in old women. Future studies should explore the molecular mechanisms involved in this response to exercise including inflammatory markers and cytokines.

### Limitations of the Study

The present study has important clinical applications; nevertheless, it also has limitations. First, the generalizability of the data obtained in this study to an expanded population is limited, because the participants were under control all day (five days a week) in a women’s rest house. Second, the participants had the same breakfast and lunch (no dietary intervention was applied); however, in other populations, this factor must be considered. Third, physical activity outside the protocol was not strictly monitored. Fourth, the study included only women; future studies should include men to determine whether similar effects are generated. Finally, trials of moderate aerobic exercise with physical fitness tests are necessary to establish their impact on physical performance.

## Figures and Tables

**Table 1:** Anthropometric and biochemical features of the study groups.

Variable	HOW (N = 60)	OW-MetS (N = 60)	
Age (years)	64.60 ± 4.90	66.80 ± 3.90	
Size (cm)	158 ± 5.50	157 ± 6.40	
Weight (Kg)	56.60 ± 4.20	68.40 ± 7.3 *	
BMI	22.71 ± 1.90	27.80 ± 3.1 *	
Waist circumference (cm)	71.08 ± 3.97	86.21 ± 5.37 *	
Body fat (%)	24.11 ± 1.13	27.19 ± 3.79 *	
Muscle mass (%)	39.85 ± 1.59	36.05 ± 2.5 *	
SBP	107.39 ± 10.50	130.96 ± 8.0 *	
DBP	73.90 ± 6.50	85.30 ± 10.7 *	
Biochemical parameters			Reference values
Glucose (mg/dL)	90.95 ± 5.47	128.31 ± 42.11 *	80–110
Total cholesterol (mg/dL)	194.60 ± 35.11	204.25 ± 31.92	<200
Triglycerides (mg/dL)	140.50 ± 15.74	211.98 ± 44.49 *	<150
HDL-cholesterol (mg/dL)	55 ± 0.34	45.00 ± 0.43 *	50–60
T/G	4.72 ± 0.06	5.10 ± 0.18 *	<4.9
TG/HDL	2.55 ± 0.28	4.70 ± 0.99 *	<3
Insulin µUI	9.9 ± 3.77	45.50 ± 17.4 *	2–10
HOMA	2.2 ± 0.85	15.30 ± 9.3 *	<2

Values are presented as means ± SD or number (%). Inter-group differences were analyzed by Student’s *t*-test * *p* < 0.05. HOW = healthy old women; OW-MetS = old women with metabolic syndrome; BMI = body mass index; SBP = systolic blood pressure; DBP = diastolic blood pressure; HDL = high-density lipoprotein; T/G = triglyceride/glucose index; TG/HDL = triglyceride/high-density lipoprotein; HOMA = homeostasis model assessment.

**Table 2:** Use of medications and supplements in study groups.

Medication/Treatment	HOW (N = 60)	OW-MetS (N = 60)
None	47 (78.3%)	0 (0)
Calcium	10 (16.7%)	0 (0)
Occasionally acetaminophen (paracetamol)	3 (5.0%)	0 (0)
Metformin	0 (0)	10 (16.7%)
Metformin + Sitagliptin	0 (0)	11 (18.3%)
Metformin + Glimepiride	0 (0)	5 (8.3%)
Metformin + Glibenclamide	0 (0)	11 (18.3%)
Metformin + Insulin	0 (0)	1 (1.7%)
Losartan	0 (0)	6 (10%)
Losartan + Hydrochlorothiazide	0 (0)	4 (6.7%)
Enalapril	0 (0)	5 (8.3%)
Amlodipine	0 (0)	7 (11.7%)
Total	60 (100%)	60 (100%)

**Table 3:** Plasmatic concentration of biomarkers of oxidative damage of the study groups.

Biomarker	HOW (N = 60)	OW-MetS (N = 60)
TBARSs (µM)	8.98 ± 4.21	11.18 ± 8.08 *
MDA (µM)	3.80 ± 0.94	5.31 ± 0.80 *
4-HNE (µM)	3.21 ± 0.69	8.85 ± 2.50 *
Carbonyl groups (nmol/mg protein)	1.46 ± 0.39	2.52 ± 0.24 *
Formazan (nmol/mg protein)	13.05± 1.12	20.80 ± 3.47 *
Thiol groups (nmol/mg protein)	5.66 ± 0.89	3.48 ± 0.85 *
GPx (µU/mg protein)	329.84 ± 117.80	298.68 ± 102.50 *

HOW = healthy old women; OW-MetS = old women with metabolic syndrome; TBARSs = thiobarbituric acid-reactive substances; MDA= malondialdehyde; 4-HNE = 4-hydroxynonenal; GPx = glutathione peroxidase. Values are expressed as mean ± SD, analyzed by *t*-Student tests; * *p* < 0.05 was considered statistically significant.

**Table 4:** Changes in anthropometric and biochemical parameters generated by the exercise program.

	HOW (N = 60)				OW-MetS (N = 60)					
Variable	Baseline	After	Change	Paired-*t*	Baseline	After	Change	Paired-*t*	*F*	
Waist circumference (cm)	71.08 ± 3.97	70.50 ± 4.10		0.87	86.21 ± 5.37	81.86 ± 5.37 *	4.35	4.35	Time	335.3
									Parameter	8486
									T × P	4628
Body fat (%)	24.11 ± 1.13	22.61 ± 0.99 *	−1.5	7.94	27.19 ± 3.79	25.80 ± 3.40 *	−1.39	2.95		
Muscle mass (%)	39.85 ± 1.59	40.65 ± 1.05 *	0.80	3.20	36.05 ± 2.50	37.15 ± 2.03 *	1.10	3		
Biochemical parameters										
Glucose (mg/dL)	90.95 ± 5.47	86.73 ± 6.30 *	−4.22	4	128.31 ± 42.11	106.73 ± 21.21 *	−21.58	4.40		
Cholesterol (mg/dL)	194.60 ± 35.11	174.45 ± 16.33 *	−20.15	4.20	204.25 ± 31.92	167.66 ± 27.72 *	−36.59	6.10		
Triglycerides (mg/dL)	140.50 ± 15.74	115.5 ± 20.32 *	−25	6.55	211.98 ± 44.49	184.1 ± 25.92 *	−27.88	4.60		
HDL-C (mg/dL)	55 ± 0.34	56 ± 0.49 *	1	14.60	45.00 ± 0.43	50.46 ± 0.96 *	5.38	9.20		
TyG	4.72 ± 0.06	4.59 ± 0.09 *	−0.13	6.60	5.10 ± 0.18	4.92 ± 0.12 *	−0.15	5.10		
TG/HDL	2.55 ± 0.28	2.04 ± 0.36 *	−0.51	6.10	4.70 ± 0.99	3.64 ± 0.51 *	−1.06	6.80		
Insulin (µU/mL)	9.9 ± 3.77	6.32 ± 3.13 *	−3.58	5.60	45.50 ± 17.40	33.5 ± 16.2 *	−12	4.10		
HOMA-IR	2.2 ± 0.85	1.35 ± 0.68 *	−0.85	5.80	15.30 ± 9.30	9.13 ± 5.13 *	−6.17	4.90		

Data are expressed as mean ± SD standard and were analyzed using a paired *t* test. The inter-group and inter-time interaction tests were performed using ANOVA mixed models. HOW: healthy old women; OW-MetS = old women with metabolic syndrome. HDL-C: high-density lipoprotein cholesterol; TyG: triglyceride–glucose index; TG/HDL-C: triglyceride-to-HDL cholesterol ratio; HOMA-IR = homeostasis model assessment-insulin resistance. Statistical significance was set at * *p* < 0.05.

**Table 5:** Changes generated in biomarkers of oxidative damage and antioxidant system by the exercise program.

	HOW N = 60			OW-MetS N = 60		
	Baseline	After	t-Value	Baseline	After	t-Value
TBARSs (µM)	8.98 ± 4.21	5.68 ± 1.20 *	6.00	11.18 ± 8.08	8.58 ± 2.66 *	2.27
MDA (µM)	3.80 ± 0.94	3.34 ± 1.21 *	2.41	5.31 ± 0.80	4.76 ± 0.63 *	3.95
4-HNE (µM)	3.21 ± 0.69	1.75 ± 0.56 *	12.55	8.85 ± 2.50	7.97 ± 5.02	1.22
Carbonyl groups (nmol/mg protein)	1.46 ± 0.39	1.27 ± 0.48 *	2.25	2.52 ± 0.24	1.09 ± 0.50 *	19.17

HOW: healthy old women; OW-MetS: old women with metabolic syndrome. TBARSs: thiobarbituric acid-reactive substances; MDA: malondialdehyde; 4-HNE: 4-hydroxynonenal. Data are expressed as mean ± SD and were analyzed using paired *t* test. * *p* < 0.05 was considered statistically significant.

## Data Availability

The original contributions presented in this study are included in the article. Further inquiries can be directed to the corresponding author.
